# An fNIRS Study of Brain Lateralization During Observation and Execution of a Fine Motor Task

**DOI:** 10.3389/fnhum.2021.798870

**Published:** 2022-01-26

**Authors:** Kosar Khaksari, Elizabeth G. Smith, Helga O. Miguel, Selin Zeytinoglu, Nathan Fox, Amir H. Gandjbakhche

**Affiliations:** ^1^National Institute of Child Health and Human Development, National Institutes of Health, Bethesda, MD, United States; ^2^Department of Behavioral Medicine and Clinical Psychology, Cincinnati Children’s Hospital, Cincinnati, OH, United States; ^3^Department of Human Development and Quantitative Methodology, University of Maryland, College Park, MD, United States

**Keywords:** mirror-neuron system, lateralization, imitation, handedness, fNIRS, action observation network (AON)

## Abstract

Brain activity in the action observation network (AON) is lateralized during action execution, with greater activation in the contralateral hemisphere to the side of the body used to perform the task. However, it is unknown whether the AON is also lateralized when watching another person perform an action. In this study, we use fNIRS to measure brain activity over the left and right cortex while participants completed actions with their left and right hands and watched an actor complete action with their left and right hands. We show that while activation is lateralized when the participants themselves are moving, brain lateralization is not affected by the side of the body when the participant is observing another person’s action. In addition, we demonstrate that individual differences in hand preference and dexterity between the right and left hands are related to brain lateralization patterns.

## Introduction

The action observation network (AON) is comprised of brain regions that are active when watching another person execute an action (Lepage and Théoret, 2006; Cross et al., 2009; Condy et al., 2021). Self-initiated motor actions using one side of the body lead to lateralized activation patterns in the brain, such that they primarily activate contralateral motor cortex with more limited activation in ipsilateral cortical regions (Colebatch et al., 1991; Rao et al., 1993; Pulvermüller et al., 1995). However, lateralization patterns in the AON when watching another person act and their relation to lateralization for self-executed actions, are unknown. Lateralization patterns of brain activation during observation of others’ actions and self-executed actions are important because they provide clues regarding the development and function of the AON. Specifically, brain lateralization patterns while watching another person’s actions may clarify neural mechanisms of imitation, an important developmental task that influences play, social development, and learning throughout the lifespan (Acharya and Shukla, 2012; Kilner and Lemon, 2013).

**Figure 1:**
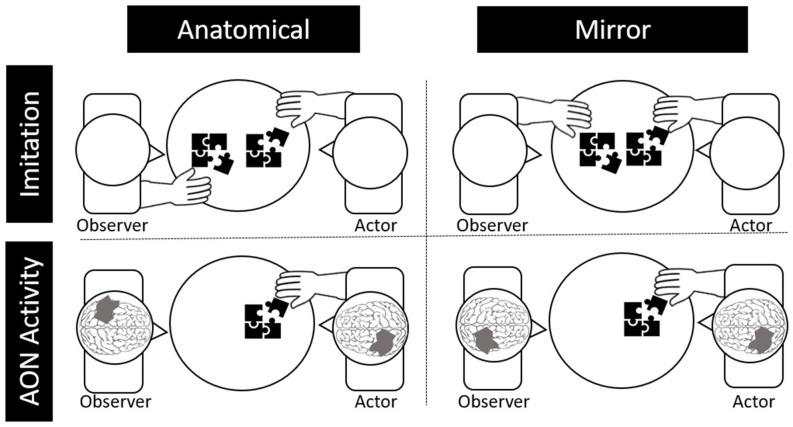
Depictions of anatomical and behavioral imitation (top) and anatomical and behavioral neural activity during observation (bottom).

Lateralization patterns in the AON during action observation could vary in the brain for imitation depending on the individual’s perception. Specifically, imitation of others’ motor behaviors can be characterized as either “mirror” imitation or “anatomical” imitation, depending on how the imitator maps their own body to the person they are imitating (Franz et al., 2007). Mirror imitation occurs when the imitator matches the side of the shared space with the other, as if they were looking in a mirror (e.g., mapping the other’s right hand to their left, see Figure 1). On the other hand, anatomical imitations occur when the imitator matches the side of their body as if they were mapping their body onto the other’s (e.g., mapping their right hand with the other’s right hand).

Likewise, activity in the AON can be either “mirror” oriented or “anatomically” oriented. Specifically, if the observed actor, who is sitting across from the observer, moves their right hand, it would lead to greater activation in the right relative to the left motor cortex for mirror representation (i.e., as if the observer were using their left hand). For anatomical representation in the brain, the same observed movement would lead to greater left relative to right motor cortex activation. Although electroencephalography (EEG) and functional magnetic resonance imaging (fMRI) are the primary methods used to study the AON, brain lateralization of the AON is challenging to study using EEG due to limited spatial resolution or using fMRI due to effects of motion artifact on the MR signal (Condy et al., 2021).

Functional near infrared spectroscopy (fNIRS) is an ideal methodology for studying lateralization of the AON for several reasons (Fukuda and Mikuni, 2012). First, compared to other neuroimaging methods, it is relatively unperturbed by motion artifacts that are intrinsic to action-observation paradigms. That is, removal of trials with motion artifact and removal of motion artifact are less likely to influence results in fNIRS (Cooper et al., 2012; Brigadoi et al., 2014; Brigadoi and Cooper, 2015). In addition, while fNIRS does not have the spatial resolution of fMRI or MEG, it relies on localizable changes in oxygenated and deoxygenated hemoglobin on the cortical surface, and thus has the resolution to detect lateralization differences and to probe the primary motor cortex. Finally, fNIRS is highly tolerated, portable, and poses minimal risk, making it ideal for use in developmental science, including studies with children in imitation or within the AON. fNIRS uses near infrared (NIR) light to measure the diffusion of photons in human tissue or cortical tissue in the case of brain imaging. Hemodynamic fluctuations in the cortex are calculated as the difference in light absorbance of oxygenated hemoglobin (HbO) and deoxygenated hemoglobin (HbR) (Sassaroli et al., 2006; Yu et al., 2006). Thus, while fMRI is the gold standard for *in vivo* imaging of the human brain, fNIRS has exceptional portability and robustness to noise along with a higher temporal resolution (Strangman et al., 2002; Cutini et al., 2012; Gagnon et al., 2012; Wilcox and Biondi, 2015; Herold et al., 2017; Pinti et al., 2018).

Although there have been several fNIRS studies of the AON, most measured activation only in the contralateral hemisphere (Condy et al., 2021) and thus could not be used for the investigation of lateralization patterns. Those fNIRS studies that did measure activation across both hemispheres either did not have action and observation conditions across both the left and right hand (Bhat et al., 2017; Crivelli et al., 2018), did not use a live person as the “actor” (Holper et al., 2010), focused on atypical populations (Kajiume et al., 2013), or used a non-lateralized action (i.e., walking; Zhang et al., 2019).

The present study therefore uses fNIRS to measure lateralization patterns in brain activity while participants: (1) complete both left- and right-handed motor actions themselves; and (2) observe both right- and left-handed motor actions of an individual sitting across from them. Observation and self-action were completed across both the left and right hand while changes in blood oxygenation levels were measured across the left and right motor cortices. In addition, we also measured hand preference and manual dexterity. We hypothesized that: (1) for the self-action condition, participants would show stronger activation in the contralateral vs. ipsilateral cortex for use of both the left and right hands; (2) laterality of brain activations for observing the actor using their left vs. right hands would vary. Specifically, activation of left and right motor cortex would show opposite lateralization patterns reflecting either mirror representation (e.g., greater left vs. right activation when the actor sitting across from them used the left hand) or anatomical representation (i.e., greater right vs. left activation when actor sitting across from them used the left hand); and (3) we expected the strength of these patterns to be positively correlated with degree of handedness. Our alternative hypothesis was that activation while observing an actor move would either: (1) activate both left and right motor cortices similarly (indicating non-lateralization of the AON for this specific task) or (2) activate the brain as if the dominant hand were being used, independent of whether the actor was using their right or left hand (indicating a handedness or experience-driven lateralization of the AON).

## Methods

This study was approved by the Institutional Review Board at the University of Maryland. All participants provided written informed consent before start of procedures.

*Participants and Measures*. Participants were 41 undergraduate students (66% female) who were recruited through undergraduate psychology courses *via* SONA (20 ± 1.6 yrs.). Six subjects were Hispanic or Latino, 11 African American, 10 white, 11 Asian, 1 white/Asian, two more than one race, and two subjects did not report their race. Thirty-eight subjects were self-reported right-handers and three were left-handed. Left-handers were excluded from these analyses since there were not enough in the left-handed group for group-based analyses. Instead, continuous measures of hand preference and differential dexterity were used to determine effects of handedness. Handedness was evaluated through use of the Edinburgh handedness inventory (Oldfield, 1971; Jin et al., 2020) while manual dexterity was evaluated with the Purdue pegboard task (Tiffin and Asher, 1948; Hannanu et al., 2020). The EHI is a dimensional hand preference score—scores range from 0 to 50, with 0 indicating the highest possible preference for the left hand and 50 indicating the highest preference for the right hand. The Purdue Pegboard (Buddenberg and Davis, 2000) task involves placing as many pegs as possible within a standardized pegboard using either the right or left hand within 30 s. Two trials of 30 s were completed per hand across two different orders to reduce practice effects across the sample.

*Self-action Task*. Participants completed simple fine motor tasks across “left” and “right” conditions while seated at a small table with a research assistant sitting opposite from them. These tasks were designed for downward expansion with young children and are similar to basic fine motor tasks that are challenging but achievable in the preschool years. The five fine motor tasks included: (1) putting plastic pennies in a bank; (2) moving small pom poms from one bowl to another with large plastic tweezers; (3) putting plastic pegs in a pegboard; (4) putting large wooden beads on a rod; and (5) turning large wooden bolts on a wooden screw. Each of these five tasks was completed across left and right hands (10 times total) for 15 s each time. Five tasks were used to maintain interest in extensions to younger age groups and to increase ecological validity. Tasks were randomly distributed across five blocks, and prior to each block the task objects (e.g., pennies and bank) were placed on the table in front of the participant and they were told whether they would be using their right or left hand. After an auditory trigger from the fNIRS interface subjects began performing the fine motor activity continuously for 15 s per trial. This resulted in five blocks of eight trials for both right and left hand.

*Observation Task*. Participants observed a trained research assistant who was sitting across from them complete the same five fine motor tasks described above with either their left or right hand. Participants were told to count the number of repetitions of each act to ensure their attention to the actor. Whereas for the Self-action task, participants were told which hand to use prior to the start of the block; for the Observation Task, the participant did not know which hand the research assistant would use but did see the task objects placed on the table in front of the research assistant. As in the self-action task, there were five trials in which the research assistant used their right hand and five trials in which they used their left, determined ahead of time through five semi-random orders. Self-action and observation trials were interleaved, as were 20 trials across two other conditions (observe next-to left and observe next-to right) which were not analyzed here. Between trials, there was a random jitter of 12–15 s of rest presented.

**Figure 2:**
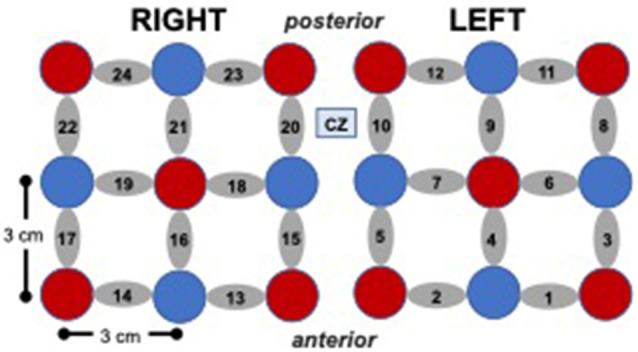
Cap array, with red circles representing light sources, blue circles represent detectors, and gray ellipses representing channels.

*fNIRS System and Probe*. Changes in oxyhemoglobin were measured using the Hitachi ETG 4000 fNIRS system with 24 channels (10 sources, eight detectors) distributed bilaterally (12 channels per side), with the most medial and anterior channels centered at CZ based on the international 10–20 electrode placement system (see Figure 2). The sampling rate was 10 Hz and source-detector separation distance was 3 cm for all the channels.

*fNIRS data Processing*. Raw Hitachi fNIRS data were processed using HOMER2 (Huppert et al., 2009) using the intensity data collected at two wavelengths (695 and 830 nm). Noisy channels (i.e., those from optodes with little to no scalp contact) were detected and removed using the HOMER2 Prune Channel function. Data were then transformed from intensity to optical density. Motion artifacts were removed using a wavelet motion correction filter of order 6. Biological (e.g., heartrate) and technical (e.g., motion) artifacts were removed using a low pass filter with a cutoff frequency of 0.1 Hz, while linear and nonlinear trends in the signals were removed by fitting a low order (an order of 3) polynomial to the fNIRS signals and subtracting it from the original signal (Huppert et al., 2009; Cooper et al., 2012; Dashtestani et al., 2019).

The processed optical density signals were then converted to oxyhemoglobin, deoxyhemoglobin, and total hemoglobin using the modified Beer-Lambert Law (mBLL). A single differential pathlength factor (DPF) of 6 was used for both wavelengths of 695 nm and 830 nm in the analysis based on the value found from previous NIRS studies on human brain (Pierro et al., 2012). We examined the NIRS signal with respect to the baseline for each subject to minimize the effects of extra-cerebral layer contamination. The source-detector distance (3 cm) was sufficient to ensure that the cerebral cortex was sampled. Additionally, previous NIRS studies have shown that the task-related effects on extra-cerebral layer hemoglobin concentration is negligible (Brigadoi and Cooper, 2015). Traces were segmented into 20-s epochs around the trigger stimulus for each trial with each epoch starting 5 s prior to each stimulus. Baseline correction corresponded to the mean HbO/HbR values from −5 to 0 s. The hemodynamic response function was then generated for each channel during each condition for each participant by averaging the response curves from all trials within a condition into a single hemodynamic curve. For each channel, the maximum change in HbO (increase in chromophore concentration) and HbR (decrease in chromophore concentration) between 5 and 20 s in response to each experimental condition (observation and execution) were computed to be used as the dependent variable in subsequent analyses. Due to a greater signal-to-noise ratio, and consistent with previous fNIRS studies, we only used the HbO signal in the remaining analyses (Yamamoto and Kato, 2002; Rahimpour et al., 2020).

*Statistical Analysis*. Statistical analysis was performed using SAS (Statistical Analysis Software) 9.4v. For each channel, the maximum change in HbO was first assessed relative to the baseline using paired *t*-tests. Reported *p* values are Bonferroni adjusted for this analysis. Following this initial analysis, a mixed model was computed using channels that showed an increase in HbO hemodynamic activity relative to baseline to examine the contrasts between conditions. Furthermore, paired *t*-test were conducted between the average hemodynamic response of channels in left hemisphere (channels 1–12) and channels on the right hemisphere (channels 12–24), to determine lateralized responses per condition. To test the relation between hemodynamic response and handedness, we computed Pearson correlation coefficients between hemodynamic response ratio and handedness scores. +. Handedness scores included the Edinburgh score (0–50, with 50 indicating the highest preference for right-handed activity; Ransil and Schachter, 1994) as well as the differential dexterity ratio as determined by (Mean number of pegs placed with right hand/ Number of pegs placed with left hand; Figure 4). Values above 1 indicate better dexterity with the right hand, while values below one indicates better dexterity with the left hand.

**Figure 3:**
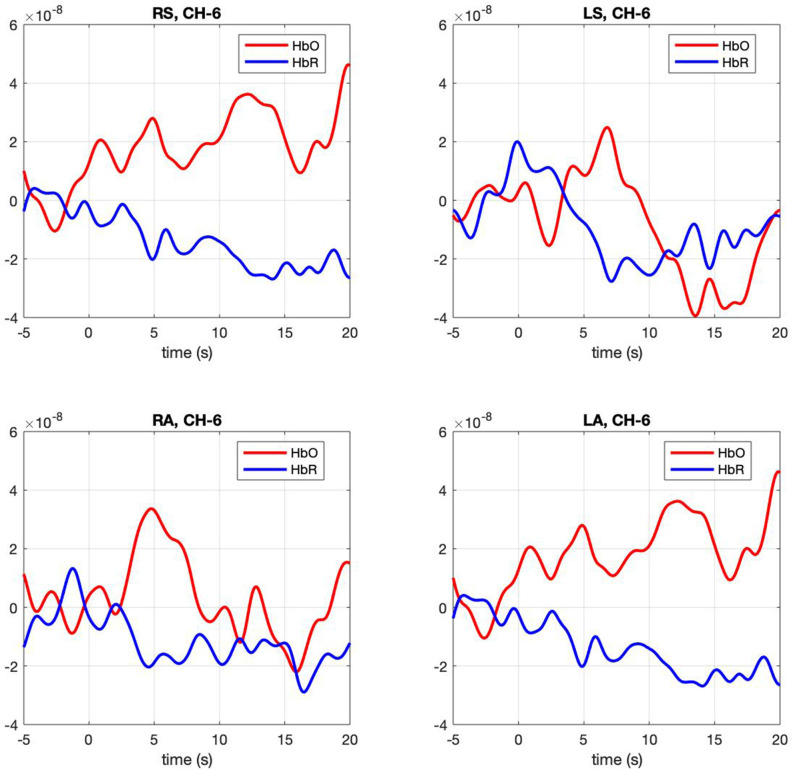
Hemodynamic response, HbO (Red) and HbR (Blue), across four conditions (RS, right self; LS, left self; RA, right across; LA, left across) across the significant channel 6.

**Figure 4:**
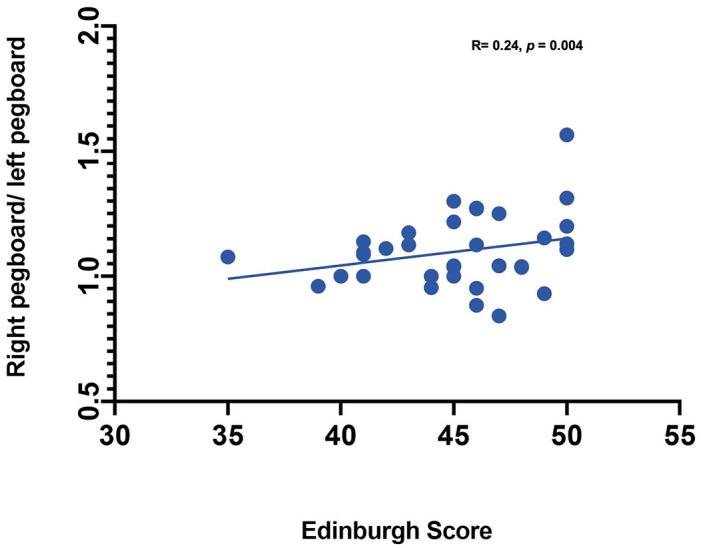
Correlation between handedness measures (Pegboard ratio and Edinburgh score).

## Results

**Contrasts between conditions.** The mixed model examining contrasts between conditions revealed a significant effect for channels 5, *F*_(3,91)_ = 2.67, *p* = 0.005 and 6 *F*_(3,91)_ = 2.67, *p* = 0.005. In both channels right-self resulted in greater hemodynamic response (*M* = 0.016, SE = 0.003 and *M* = 0.011, SE = 0.001) than left-self (*M* = 0.01, SE = 0.003 and *M* = 0.005, SE = 0.0012), right across (*M* = 0.01 SE = 0.025 and *M* = 0.006 SE = 0.001) and left across (*M* = 0.013 SE = 0.003 and *M* = 0.006 SE = 0.001), respectively. Channel 6 hemodynamic response is shown in Figure 3. The statistical significance of maximum oxyhemoglobin activation was determined against baseline using a t-test.

**Lateralization of brain activity.** Paired t-tests revealed a greater hemodynamic response in the left hemisphere for the right-self condition (*t*_(32)_ = 2.40, *p* = 0.022). No significant results were found for left-self, right-across, and left-across.

**Figure 5:**
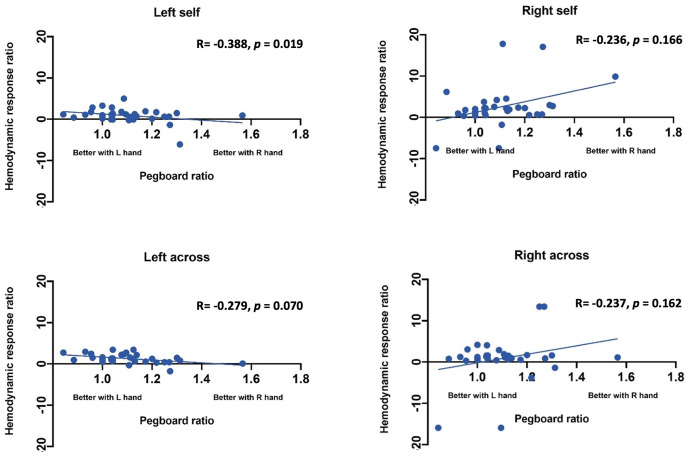
Correlation between ratio of hemodynamic response function and handedness measured by the Purdue Pegboard test.

**Figure 6:**
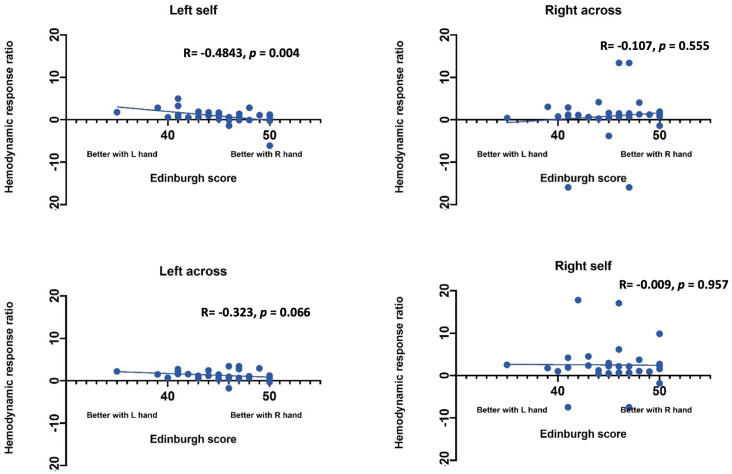
Correlation between ratio of hemodynamic response function and handedness measured by the Edinburgh handedness inventory.

**Effect of handedness**. The results from Pearson correlations indicated that there was a significant negative correlation between the L/R ratio for the left-self condition and handedness. Specifically, the correlation was significant when handedness was measured using Purdue pegboard (*rs*_(X)_ = −0.484, *p* = 0.0040; Figure 5), and Edinburgh handedness inventory (*rs*_(X)_ = −0.388, *p* = 0.019; Figure 6). No significant results were found for right-self, right-across and left-across.

### Role of Handedness vs. Hand Used in Brain Lateralization

GLM with hand used predicting lateralization ratio was significant (*t*_(35)_ = 2.28, *p* = 0.029). Specifically, in the self-condition, using the right hand was associated with a higher left, over right ratio than using the left hand. However, in the observer condition, the hand used by the other did not predict brain lateralization (*t*_(35)_ = 0.861, *p* = 0.395).

We also used GLM to determine if hand preference predicts brain lateralization in the self-condition above and beyond what is predicted by whether the participant uses their right or left hand. Hand preference did not predict brain lateralization above and beyond the side used in the self-condition (*t*_(35)_ = −1.16, *p* = 0.25). Differential dexterity also did not predict brain lateralization above and beyond side in self condition (*t*_(35)_ = −0.031, *p* = 0.976). Hand preference also does not predict brain lateralization in the observe condition (*t*_(35)_ = −0.691, *p* = 0.494). However, differential dexterity did predict brain lateralization in the other condition (*t*_(35)_ = −2.167, *p* = 0.0375). Specifically, individuals who were more dexterous with their right hand showed less left lateralized brain activity when watching the actor use both their left and right hands.

## Discussion

In this article, we showed that lateralization of activity in the action observation network varies for self-initiated actions and for observed actions. Specifically, using your right vs. left hand led to increased leftward lateralization of brain activity in the self-condition, whereas lateralization of brain activity did not vary when observing the other use their right vs. left hands. We demonstrated that channels placed in the left motor cortex (approximately) are specifically activated by use of the right hand during self-actions (vs. all other conditions). We also show that hand preference and differential dexterity are related to lateralization of the AON across both self and observe conditions. Specifically, we show that while hand used by the person being observed does not drive lateralization of brain activity in the observer, brain lateralization patterns are related to the patterns of hand preference and dexterity. Specifically, preferring the right hand and being better with the right hand were both associated with decreased leftward lateralization when acting with the left hand. Also, having higher dexterity with the right vs. left hand was associated with decreased leftward lateralization during the observe condition independent of which hand the actor was using.

Although mirror vs. anatomical representation have not been directly studied in the AON, multiple studies have shown differential lateralization patterns in the brain for action execution vs. observation. One pattern that has been seen across multiple studies is that when using right-handed subjects, there is greater left hemisphere activation in motor regions regardless of whether the observed action is with the left or right hand (Koski et al., 2002). This is very much in line with our findings, and suggests that once handedness has emerged, brain lateralization patterns associated with handedness are an important determinant of neural mirroring patterns. Extrapolated to mapping others’ actions, this supports the possibility that observers activate brain regions “as if” they were doing the task with their dominant hand. It is unclear how these patterns would present in younger children with less lateralized motor behaviors and completing this research in young children would clarify how handedness may drive development of neural mirroring and/or imitation abilities.

Importantly, individuals’ handedness is also known to affect brain lateralization. Specifically, the ratio of contralateral to ipsilateral activation when using the dominant hand has shown a linear association with degree of handedness (Dassonville et al., 1998). The present study replicates that pattern and extends it beyond hand preference to differential dexterity as well. In addition, we show that handedness is related not only to brain lateralization for the individual during their own actions but also to brain lateralization when observing others. This provides further support to the idea that activation in the action observation network is tied to an individual’s own experiences and expertise (Condy et al., 2021).

While this study is the first to examine brain lateralization across both self and other conditions and with the left and right hand, several limitations should be noted. First, we did not include left handers in this study due to low numbers recruited. While only 10% of humans are left-handed, their brain lateralization in the AON is still meaningful and needs to be studied. For example, we found here that individual handedness was related to AON activity even when observing others, but it is possible that this would not be true in left handers, which could alter the interpretation of these results. Second, while we placed the optode array in relation to the 10–20 international classification system, individuals’ heads can vary and therefore we cannot clearly determine exact neuroanatomical locations of hemodynamic activity. Future studies should include digitizing methods to determine the precise location of each channel would allow for determination of activity in primary motor regions vs. somatosensory cortex. In addition, we used a newly designed motor task that was designed to be interesting enough for toddlers and preschoolers to tolerate. Since infant tasks and adult tasks typically rely on very repetitive actions, we included five different tasks that were of sufficient difficulty to be interesting to a toddler or preschooler. While our task clearly elicited lateralized activity during the self-action condition, it is possible that lack of lateralization during observation was task specific, which indicates the need for replication. Finally, the observe condition for the present study involved having the participant sit across from the person that they were observing. However, it is possible that lateralization of brain response (and thus, mirror vs. anatomical activation) would vary if the participant were sitting next to the actor. Therefore, future studies should clarify the role of actor orientation in lateralization of AON activity.

## Data Availability Statement

The raw data supporting the conclusions of this article will be made available by the authors, without undue reservation.

## Ethics Statement

The studies involving human participants were reviewed and approved by The Institutional Review Board for the University of Maryland. The patients/participants provided their written informed consent to participate in this study.

## Author Contributions

The authors confirm contribution to the article as follows. Study conception and design: ES, NF, and AG. Data collection: ES. Analysis and interpretation of results: KK, HM, ES, SZ, NF, and AG. Draft manuscript preparation: KK, ES, HM, and SZ. All authors reviewed the results and approved the final version of the manuscript. All authors contributed to the article and approved the submitted version.

## Conflict of Interest

The authors declare that the research was conducted in the absence of any commercial or financial relationships that could be construed as a potential conflict of interest.

## Publisher’s Note

All claims expressed in this article are solely those of the authors and do not necessarily represent those of their affiliated organizations, or those of the publisher, the editors and the reviewers. Any product that may be evaluated in this article, or claim that may be made by its manufacturer, is not guaranteed or endorsed by the publisher.
